# Water-in-bisalt electrolytes with mixed hydrophilic and hydrophobic anions for enhanced transport and stability for potassium-ion batteries†

**DOI:** 10.1039/d4ra08378d

**Published:** 2025-01-02

**Authors:** Mukhilan Dhasarathaboopathy, Palani Sabhapathy, Burcu Gurkan

**Affiliations:** a Department of Chemical and Biomolecular Engineering, Case Western Reserve University Cleveland OH USA beg23@case.edu

## Abstract

Water-in-salt electrolytes provide an expanded electrochemical potential window, thus enabling a wide range of battery chemistries based on readily available salts and water. This study introduces a binary salt approach for achieving high K^+^ concentration with a tunable solvation sphere composed of acetate (Ac^−^) and trifluoromethane sulfonate (OTf^−^) anions, and water. Combining the hydrophilic low-cost potassium acetate with hydrophobic potassium trifluoromethane sulfonate salts, 36 molal liquid electrolyte, K(Ac)_0.9_(OTf)_0.1_·1.5H_2_O, is achieved with an electrochemical stability window spanning from −1.74 V on the Al electrode to over 3 V on the Ti electrode, exceeding a total of 4.74 V and an ionic conductivity of 18 mS cm^−1^ at 25 °C. Both the Raman and NMR analyses show strong water-Ac^−^ interactions within the primary solvation shell of K^+^. In parallel, OTf^−^ is found to be outside of this shell disrupting the water O–H network, thus pushing water into the K^+^ solvation shell. Elimination of the free water molecules and the solvation disproportion resulting from the bi-salt approach underlie the enhanced transport properties and the electrochemical stability window. Therefore, the mixed bi-salt approach in water-in-salt electrolytes can be a promising and cost-effective solution for advancing potassium batteries.

## Abstract 2

Enabled by the fine tuning of the potassium ion solvation through water-in-bisalt approach, wide electrochemical stability and enhanced ionic conductivity are simultaneously achieved in a 36 molal electrolyte suited for potassium batteries.

## Graphical abstract

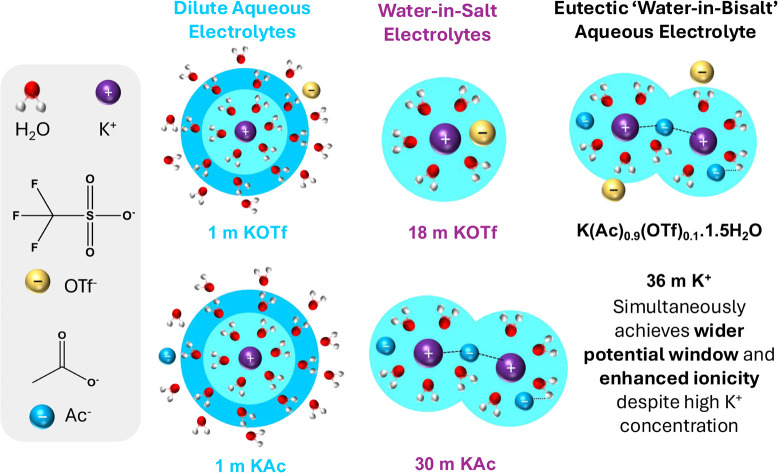

## Introduction

High-energy density batteries such as Li-ion that power portable electronics and electric vehicles rely on materials that are relatively scarce^1^ and organic volatile electrolytes that are flammable.^2^ In regards to the electrolyte challenge, the aqueous systems have a limited electrochemical stability window (ESW) of about 1.23 V,^3^ and therefore not suited for high-voltage electrode materials. Recently, the ESW of aqueous electrolytes has been successfully widened by increasing the Li-salt concentration. In 2015, Suo *et al.* introduced the “water-in-salt” electrolyte (WiSE) concept, demonstrating an ESW expansion to 3 V on stainless steel using a highly concentrated lithium bis(trifluoromethanesulfonyl)imide solution.^4,5^ It is believed that in WiSE, where the salt concentration surpasses that of water, all water molecules are bound within the ion solvation shell, thus reducing water activity.^6^ This approach and the later-developed “water-in-bisalt” (WiBS) concept involving dual salts have significantly increased the ESW of aqueous electrolytes. In these concentrated systems involving Li-salts, all water molecules participate in the Li^+^ solvation shell and the anion-derived protective film further contributes to enabling high energy density in aqueous Li-ion batteries.^5,7,8^ In regards to the material scarcity challenge, earth abundant alternatives involving sodium (Na) and potassium (K) based systems have been of interest.^9^ The highest reported salt concentrations for room-temperature hydrate melts in these systems are 35 m for Na-ion^10^ and 61.7 m for K-ion,^11^ typically employing hydrophobic anions. Recent studies have also explored hydrophilic anions such as acetate (Ac^−^) in K-salt systems.^6,12^ WiBS electrolytes have also been reported, however they remained to have either hydrophobic or hydrophilic anions with different cations (*e.g.*, K^+^, Li^+^).^6,8,11^ Further, the prior WiBS, despite achieving higher salt concentrations with wider ESWs, ionic conductivities were lowered with increased viscosities, in comparison to WiSE.

Recognizing these challenges, we explored the role of salt anions in influencing the physical and chemical properties, focusing on how they integrate into solvation structures and interact with water molecules. In particular, the combination of hydrophobic anions that are key in liberating ‘free’ water molecules, thus lowering bulk viscosity and hydrophilic anions that strongly interact with water molecules through hydrogen bonding, thus enhancing salt solubility is considered. Through the mixtures of potassium acetate, KAc, (hydrophilic) and potassium trifluoromethane sulfonate, KOTf, (hydrophobic) salts, we developed a eutectic mixture that achieved 36.1 m K^+^ with simultaneously enhanced ionic conductivity and ESW, superseding the parent aqueous WiS based on a single salt. Through the Raman and NMR spectral analyses, the solvation structure as a function of Ac^−^ : OTf^−^ ratio and K^+^ concentration is described to rationalize the measured transport properties and the electrochemical stability.

## Results and discussion

**Fig. 1:**
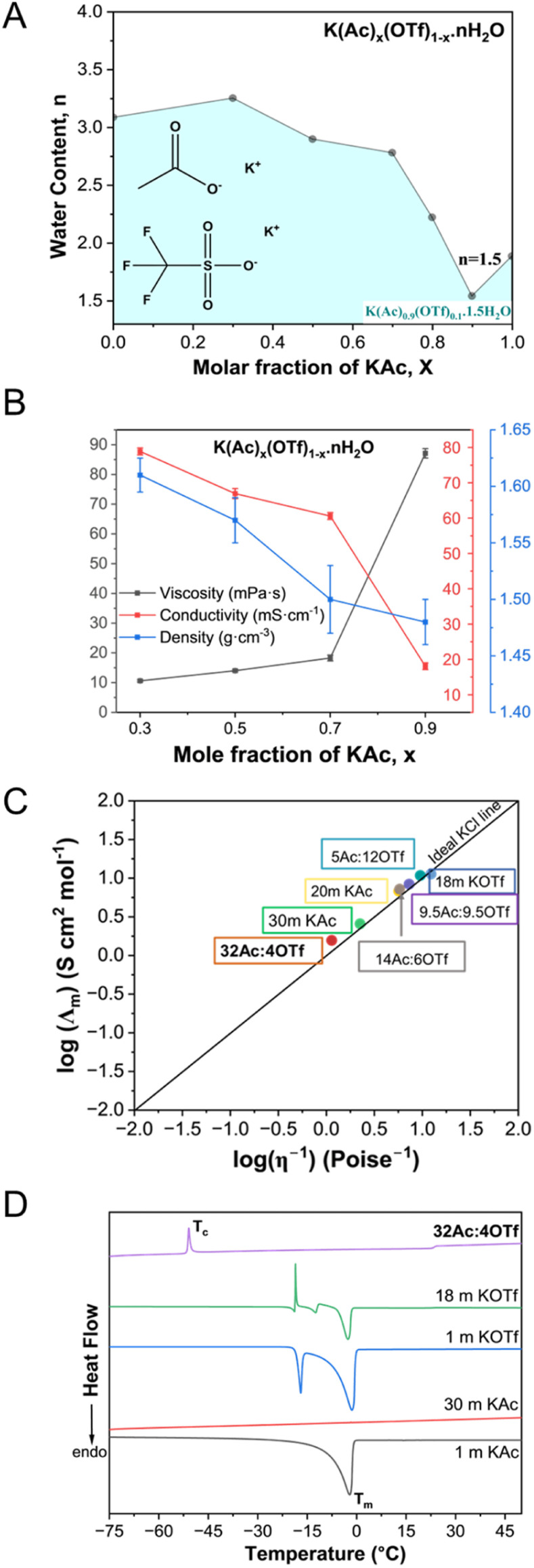
Measured properties of K(Ac)_*x*_(OTf)_1−*x*_·*n*H_2_O (*n*: H_2_O/K molar ratio): (A) liquidus phase diagram at 25 °C; (B) viscosity, ionic conductivity, and density as a function of KAc mole fraction, *x* at 25 °C; (C) Walden plot for aqueous KAc and KOTf electrolytes, in comparison to the WiBS examined. For simplicity, the mixtures are labeled according to the molality and the anion ratio, simplified as Ac^−^ : OTf^−^; (D) differential scanning calorimetry (DSC) curves of aqueous KAc and KOTf electrolytes, in comparison to the WiBS, K(Ac)_0.9_(OTf)_0.1_·1.5H_2_O labeled as 32Ac : 4OTf. DSC was collected at a rate of 1 °C min^−1^.

As seen in Fig. 1A, a eutectic composition was identified for the KAc/KOTf salt mixtures demonstrating the maximum K^+^ concentration at a KAc mole fraction of 0.9 with approximately 1.5 water molecules per cation. This WiBS, denoted as K(Ac)_0.9_(OTf)_0.1_·1.5H_2_O, corresponds to 36.1 molal K^+^. Fig. 1B shows the composition dependent density, viscosity, and ionic conductivity measured at 25 °C (tabulated data in Table S1†). Increasing salt concentration results in an increase in viscosity and a decrease in conductivity, particularly sharply passed 0.7 mole fraction of KAc. Despite these trends, the eutectic WiBS, K(Ac)_0.9_(OTf)_0.1_·1.5H_2_O, exhibits a conductivity of 18.07 mS cm^−1^ and a viscosity of 87.1 mPa s; both of these values are superior to the previously reported WiBS, such as the mixture of 32 m KAc and 8 m LiAc with a reported ionic conductivity of 5.3 mS cm^−1^ and a viscosity of 374 mPa s.^6^ Compared to more conventional electrolytes, the specific advantage of WiBS, besides enhanced safety due to the elimination of volatile components, is the superior conductivity^13^ (*e.g.*, solid-state electrolytes and organic electrolytes based on potassium salts such as KFSA, KTFSA, KPF_6_, KClO_4_ and KBF_4_ dissolved in propylene carbonate – all below 8 mS cm^−1^ at 25 °C).^14^

The Walden analysis, shown in Fig. 1C, categorizes K(Ac)_0.9_(OTf)_0.1_·1.5H_2_O as a “superionic” solution. This behavior is characterized by high ionic conductivity decoupled from viscosity.^7,11^ The categorization of the eutectic WiBS as superionic, despite the measured decrease in conductivity with increasing macroscopic viscosity (Fig. 1B), could be due to the “jumping” mechanism for K^+^ becoming favorable at extreme concentrations, likely supported by the weak Lewis acidity of K^+^. Additionally, the presence of the hydrophobic OTf^−^ alters the solvation environment; facilitating easier movement of K^+^ from one site to another, thereby enhancing ion mobility. In the perspective article by Han *et al.*,^15^ analyzing the anion effects in transport properties of highly concentrated aqueous electrolytes, they present the argument of hydrophilicity leading to homogenous systems with cooperative motions of ions (*e.g.*, jumping) whereas the hydrophobicity facilitating vehicular motion due to solvation disproportion. With the WiBS approach here, these two mechanisms can be further tuned; however, it is difficult to quantify these individually to determine their contributions to the overall macroscopic properties measured here. Therefore, we resorted to analyze the solvation environment to better understand the origins of the superionic nature.

The thermogravimetric analysis (TGA) performed between 25 and 300 °C (Fig. S1†) confirms the electrolyte composition upon preparation of the mixtures. As tabulated in Table S2,† the salt composition as determined from the weight loss (due mostly to water evaporation) agrees with the stoichiometric ratios of salt to water in the prepared samples. The differential scanning calorimetry (DSC) was employed to examine the phase transitions in WiBS in comparison to the WiSE as shown in Fig. 1D. The 1 m KAc solution exhibited a melting transition at −2.1 °C similar to low-concentration solutions,^6^ while the 30 m KAc solution displayed an amorphous nature, lacking distinct melting peaks. In contrast, both 1 m and 18 m KOTf solutions showed two endothermic peaks corresponding to the eutectic arrest and the liquidus point, with the latter also exhibiting an exothermic peak (*T*_c_) upon reheating, indicative of cold crystallization. This indicates a semi-crystalline behavior which was also observed in binary mixtures of hydrophobic anions.^16^ The eutectic WiBS, K(Ac)_0.9_(OTf)_0.1_·1.5H_2_O, remained stable as a liquid at room temperature, exhibiting a *T*_c_ at −50.9 °C lower than the aqueous 18 m KOTf (the most concentrated WiS possible for this salt). Unlike sharp melting transitions, the WiBS showed a minor transition around 23 °C corresponding to melting (*T*_m_), confirming its predominantly amorphous liquid nature with a cold crystallization.

**Fig. 2:**
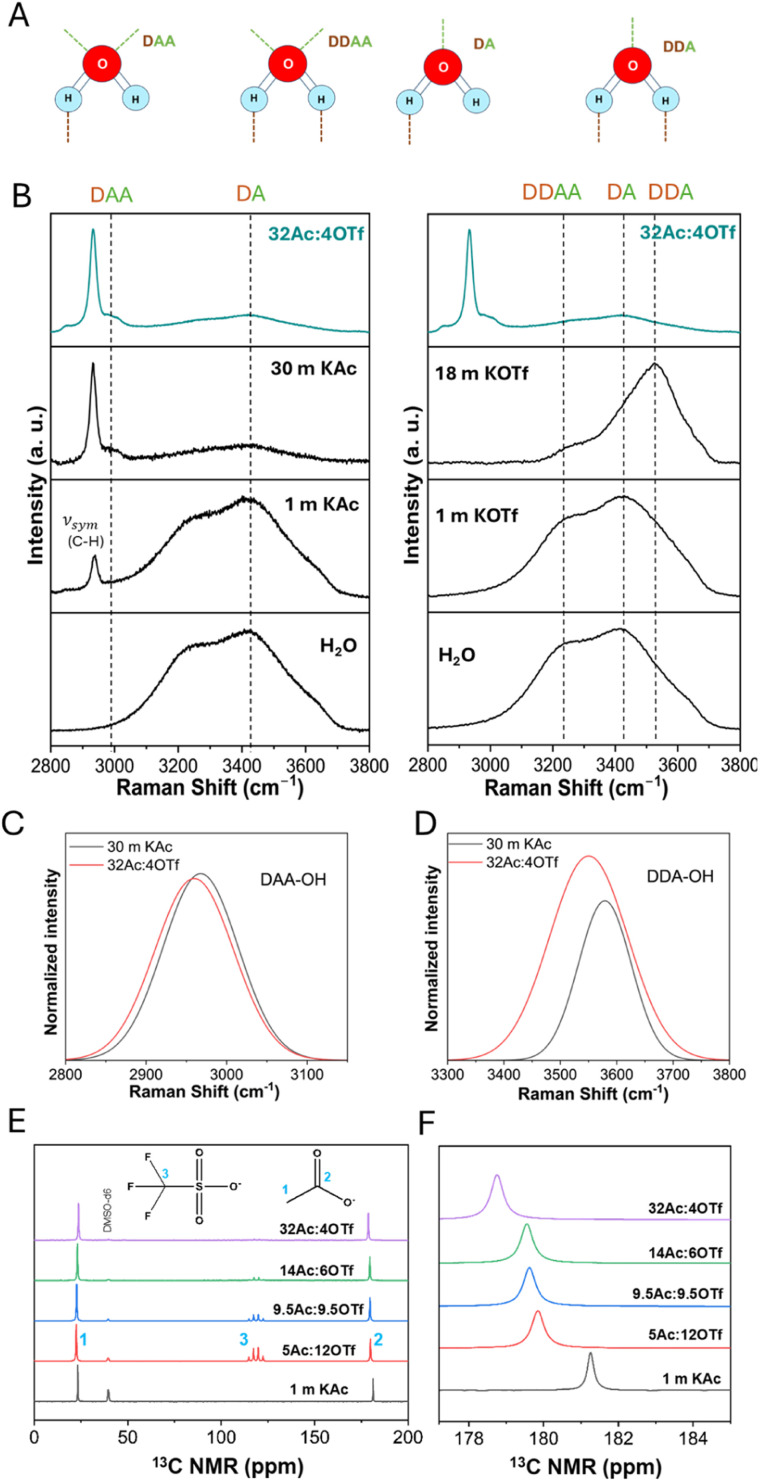
Spectral analysis of solvation and ionic interactions: (A) vibrational modes of water; (B) local Raman spectra of K(Ac)_0.9_(OTf)_0.1_·1.5H_2_O compared with 1 m and 30 m KAc aqueous solutions (left) and 1 and 18 m KOTf solutions (right); (C) DAA-OH stretching peaks of 30 m KAc and WiBS; (D) DDA-OH stretching peaks of 30 m KAc and WiBS; (E) ^13^C NMR spectra of 1 m KAc in comparison to other mixtures; (F) ^13^C NMR spectra of the carboxylate carbon of Ac^−^.

To understand the solvation structure and ion interactions, Raman and NMR analyses were performed. In particular, the 2800–3800 cm^−1^ local Raman region relevant to the O–H stretching vibration modes of water (Fig. 2A) were examined (Fig. 2B). The broad O–H stretching band of water was deconvoluted (Fig. S2–S4† for aqueous KAc, KOTf, and WiBS) into five known vibrational modes, namely DAA, DDAA, DA, and DDA of water network, and free OH symmetric stretching,^17^ corresponding to the peaks at approximately 3000, 3300, 3400, 3550, and 3650 cm^−1^, respectively. In these modes, D stands for the donor and A stands for the proton acceptor in a hydrogen bond. In KAc solutions, the hydrophilic Ac^−^ forms strong hydrogen bonds with water molecules as evident from the diminished water network vibrations (Fig. 2B, left). As the concentration of KAc increases, the intensity of the DAA peak also increases as seen in Fig. S5† where the intensity of water vibrations as determined from deconvolutions are plotted as a function of KAc concentration (those for WiBS is shown in Fig. S6†). This is in contrast to the behavior of other water network vibrations. Therefore, the DAA peak, located around 3000 cm^−1^, is primarily linked to water-Ac^−^ hydrogen bonding, where water molecules are increasingly captured by Ac^−^ through the carboxylic group. In the case of the concentrated KOTf solution (18 m KOTf), a new peak at 3535 cm^−1^ appears, corresponding to the DDA mode (Fig. 2B, right). This is indicative of an ordered arrangement of water molecules around K^+^ as the hydrophobic OTf^−^ disrupts the water hydrogen bonding network and pushes the water molecules to participate in K^+^ ion solvation. This feature is characteristic of crystalline hydrates,^18^ indicating that most water molecules are involved in the hydration of K^+^, forming a weak yet ordered DDA hydrogen bonding network.

In the WiBS, K(Ac)_0.9_(OTf)_0.1_.1.5H_2_O with low water content, the Raman spectra show a decrease in all O–H peak intensities except for the DAA peak, similar to the 30 m KAc, indicating strong water–acetate interactions (Fig. 2C). The absence of the free O–H peak (would normally appear around 3650 cm^−1^) confirms the lack of free water molecules, a characteristic of WiSE with reduced water activity (Fig. S4d†). This confirms that all water molecules present in the WiBS are incorporated into the K^+^ solvation shell. On the other hand, the DDA peak shows higher intensity in WiBS compared to 30 m KAc (Fig. 2D), suggesting that the hydrophobic OTf^−^ significantly influences ion solvation by pushing the water molecules to solvate K^+^, similar to what is observed in the 18 m KOTf solution. This peak is redshifted and broadened in the WiBS, indicating the formation of a strong but less ordered DDA hydrogen bonding network around K^+^.

To investigate the presence of anions in the K^+^ solvation shell and potential ion pairing, we analyzed Ac^−^ interactions through a series of dilutions with 1 m KAc and aqueous KAc/KOTf mixtures using ^13^C NMR spectroscopy (Fig. 2E). Fig. S7† shows the concentration dependent ^13^C NMR spectra of aqueous KAc solutions as a reference. As the Ac^−^ concentration increases, we observed an up-field shift in the carbon signal associated with the carboxylate group of the Ac^−^ (Fig. 2F). This shift suggests that more Ac^−^ coordinate with the same K^+^, increasing ion shielding and this can be due to the significant overlap of solvation shells at high concentrations. The primary coordination sphere of K^+^ now includes both water molecules and Ac^−^, with close proximity of cations to the carboxylate carbon increasing electron density, thus leading to the observed up-field shift in the NMR. The relatively narrow line widths in the ^13^C NMR spectra across all electrolyte samples point to a rapid exchange between anions and other molecules within the solvation shell, due to the weak Lewis acidity of K^+^. A slight increase in linewidths at high concentrations hints at complexation and stronger interactions between K^+^ and Ac^−^. In contrast to WiBS, ion pairing between K^+^ and OTf^−^ is seen at high concentrations in KOTf/water mixtures (Fig. S8a†), where the SO_3_ symmetric stretching peak shifts to a higher wavenumber as the OTf^−^ concentration increases. Similar shift becomes apparent in KAc/KOTf mixtures when KOTf concentration increases to 6 m (14Ac : 6OTf; Fig. S8b†). Beyond this concentration, the peak remains constant, indicating that ion-pair formation reaches a saturation point. In the WiBS, with 4 m KOTf, it can be derived that the OTf^−^ is outside the 1st solvation shell representing a solvent-separated ion pair.

**Fig. 3:**
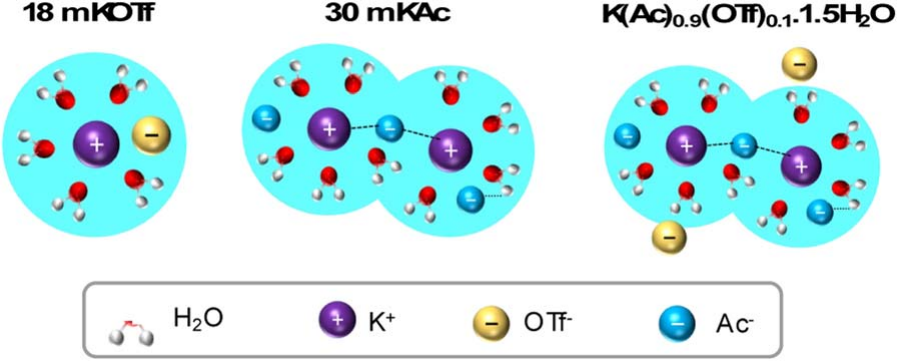
Illustration of K^+^ solvation in WiSE (KAc and KOTf) and the eutectic WiBS.

The K^+^ solvation structure in the as-prepared electrolytes, based on insights from Raman and NMR analysis, is illustrated in Fig. 3. The hydrophobic OTf^−^ ion disrupts the water hydrogen bonding network, leading to the formation of a relatively weak and less ordered DAA network around K^+^. Additionally, the rapid exchange of ions within the solvation shell supports the “jumping” mechanism described in the Walden plot, further contributing to increased ion mobility despite the very high K^+^ concentration.

**Fig. 4:**
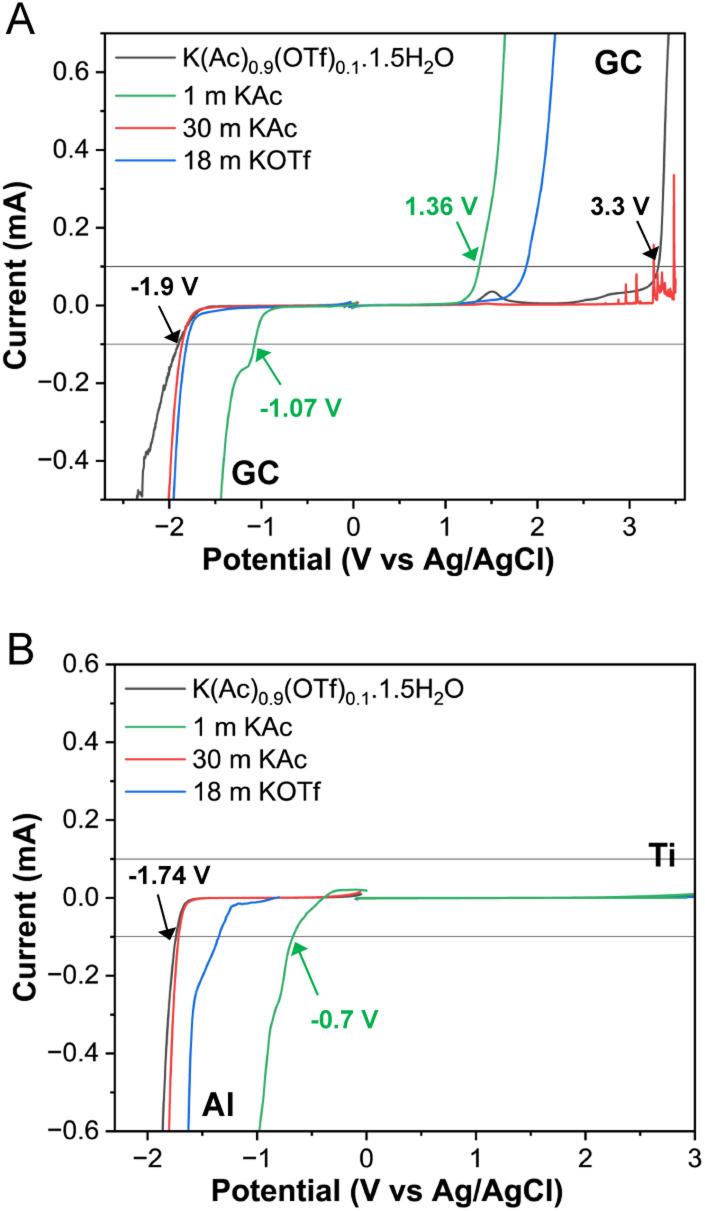
Linear sweep voltammetry (LSV) profiles recorded at a scan rate of 1 mV s^−1^ for the solutions K(Ac)_0.9_(OTf)_0.1_·1.5H_2_O, 1 m KAc, 30 m KAc, and 18 m KOTf using a glassy carbon, GC, electrode (A) and Ti and Al foils (B) as working electrodes. The dotted lines indicate the potentials at which hydrogen and oxygen evolution reactions start occurring, defining the potential window.

The electrochemical stabilities of KAc and KOTf based WiSE, and the eutectic WiBS were examined by linear sweep voltammetry (LSV) on a variety of electrode materials as shown in Fig. 4. A glassy carbon electrode was used as a comparison (Fig. 4A). The WiBS showed a much wider potential window (over 5 V) compared to 1 m KAc solution (2.4 V), due to reduced water activity. In concentrated KAc, the carbon electrode dissolved before oxygen evolution due to high alkalinity. However, adding KOTf to form the WiBS reduced alkalinity, preventing carbon corrosion and extending the potential window on the positive side. The cathodic side also showed a slight increase in potential for the WiBS.

Following previous reports relevant to the K-ion batteries,^11,19^ we used aluminum (Al) and titanium (Ti) as negative and positive electrodes, respectively. Both electrode materials demonstrated high overpotentials for water splitting (Fig. 4B), effectively suppressing hydrogen and oxygen evolution over a broad potential range. During the cathodic scan, using an Al electrode, the hydrogen evolution was significantly suppressed in both the 30 m KAc (−1.71 V *vs.* Ag/AgCl) and the WiBS (−1.74 V *vs.* Ag/AgCl) compared to the dilute 1 m KAc (−0.7 V *vs.* Ag/AgCl). All solutions showed similar behavior with the Ti electrode, exceeding 3 V *vs.* Ag/AgCl. The positive potential range in the LSV profiles lacked distinct electrochemical features, due to surface passivation of the current collector caused by anodic oxidation. The LSV curves for the first three sweeps using Al and Ti electrodes (Fig. S9†), shows a small decrease in stability window after the first voltage scan. This could be attributed to the reduction of the native aluminum oxide layer during the initial scan. In subsequent sweeps, the presence of oxide available for reduction is reduced. In comparison, the electrochemical stability gets broader with the glassy carbon electrode as the LSV is repeated. This is likely due to the formation of an anion-driven passivation layer, as discussed in prior studies.^20^ Since a potential window of over 4.7 V is achieved using Al–Ti electrodes; these material can serve as current collectors for practical electrode materials like Prussian blue analogs (K_1.67_Mn_0.65_Fe_0.35_[Fe(CN)_6_]_0.92_·0.45H_2_O) for cathode^21^ and 3,4,9,10-perylenetetracarboxylic diimide (PTCDI) for anode.^22^

## Conclusions

In summary, a eutectic WiBS electrolyte, K(Ac)_0.9_ (OTf)_0.1_·1.5H_2_O, with a high salt concentration (36 m), excellent ionic conductivity (18.07 mS cm^−1^), and a wide ESW (>4.74 V, with −1.74 V on Al and >3 V on Ti), suited for K-ion batteries, is reported. The unique combination of hydrophilic and hydrophobic anions improves both salt solubility and ion mobility while simultaneously achieving voltage stability. The absence of free water molecules due to the strong interaction of Ac^−^ with water and the redistribution of water towards K^+^ solvation driven by the hydrophobic OTf^−^ enhanced electrochemical stability, while maintaining significant ion mobility. Future studies should further investigate the interfacial behavior of WiBS developed in this study in K-ion batteries.

## Experimental methods

### Preparation of electrolytes

Potassium acetate (99.0% anhydrous) and potassium trifluoromethanesulfonate (98% anhydrous) were obtained from Sigma Aldrich and Fisher scientific respectively. Inside an argon filled glovebox (VTI Super, O_2_ < 1 ppm, H_2_O < 1 ppm), precise amounts of these salts were placed in 20 mL glass vials and sealed using crimp caps with septa, covering a concentration range from 1 m to 30 m for KAc and 1 m to 18 m for KOTf. Distilled water (Fisher) was then added using a syringe under ambient atmosphere. Higher concentration solutions were heated with stirring at 60 °C for 3 hours. Subsequently, the solutions were left overnight at room temperature (25 °C) to ensure complete dissolution of the salts.

### Liquidus line measurements

To determine the liquidus line of K(Ac)_*x*_(OTf)_1−*x*_·*n*H_2_O salt/water mixtures, varying mole fractions of KAc(*x*) and KOTf (1−*x*) were weighed inside an Argon-filled glovebox (VTI Super, O_2_ <1 ppm, H_2_O < 1 ppm), starting from *x* = 0 to *x* = 1. Water was incrementally added to each vial using a volumetric glass syringe. Subsequently, the vials underwent approximately one hour of heating at around 60 °C after each water addition. This process of sequential water addition and heating was repeated until complete dissolution of the salt mixture was achieved upon cooling to room temperature. The solutions were left overnight at near room temperature (25 °C) to ensure complete dissolution of the salts.

### Physicochemical properties

Density (*ρ*/g cm^−3^) measurements for the KAc/H_2_O, KOTf/H_2_O and the KAc/KOTf/H_2_O were performed using a U-tube density meter (DMA-T500, Anton-Paar, ±0.01 mg mL^−1^) at 25 °C. Instrument calibration was monitored by using air and distilled water before each experiment. The standard uncertainty associated with the densitometer is estimated to be *u*(ρ) = 0.00005 g cm^−3^, *u*(*T*) = 0.03 °C. The standard uncertainty associated with the density measurements, calculated from three sets of samples, is ±0.03 g cm^−3^. Viscosity for these samples were measured using the RheoSense MicroVISC microchannel viscometer within a RheoSense MicroVISC Temperature Control unit, maintaining precision within ±0.10 °C, with a sample flow rate of approximately 0.75 μL s^−1^. A single measurement required approximately 10–20 μL of sample. The viscometer was calibrated using certified viscosity reference standards from Cannon Instrument Company. A dual platinum electrode cell (MMA 500, Materials Mates Italia) with a cell constant of 1.01 cm^−1^ (0.1 M KCl reference) was used to measure ionic conductivity, and a BioLogic SP-200 potentiostat with a frequency response analyzer covering 7 MHz to 10 μHz was used for electrochemical impedance spectroscopy. The cell was placed within the RheoSense MicroVISC Temperature Control unit (±0.10 °C) to regulate the temperature for conductivity measurements. The intercept of a linear fit to the capacitive region of the Nyquist plot (imaginary *versus* actual impedance) was used to calculate the solution resistance. *u*(*σ*) = 0.05 is the estimated standard uncertainty.

### Thermogravimetric analysis (TGA)

TGA (Discovery TGA 55) was performed for the WiS electrolytes including the binary mixtures to confirm the salt weight percentage and to examine the thermal decomposition. Thermal decomposition (Td) was measured by employing a 10 °C min^−1^ ramp starting from 25 to 300 °C with equilibrating it at 25 °C before ramping.

### Differential scanning calorimetry (DSC)

By using differential scanning calorimetry (DSC3, Mettler Toledo), phase transitions were identified. First, samples (10–15 mg) underwent one heating/cooling cycle at a pace of 10 °C min^−1^, going from −80 °C to 80 °C to −80 °C. The sample was annealed at −80 °C for 5 minutes. Next, the sample was heated at a rate of 1 °C min^−1^ from −80 °C to 80 °C. Starting at the maximum point of the endothermic peak, the melting point (Tm) was determined.

### Raman spectroscopy

Raman spectroscopy was employed to examine the liquid structure and coordination states of the electrolyte mixture at near room temperature (25 °C). Utilizing a confocal Raman microscope (InVia, RENISHAW), spectra were captured for the whole spectral range from 200–3800 cm^−1^ and then localized within the spectral range of 2800–3800 cm^−1^ for understanding water–water interactions. A 514 nm HeNe laser served as the excitation source, with each spectrum recorded using 20x magnification objective, over a 15 seconds exposure time and 3 accumulations with 50% laser power. Fityk 1.3.1 software was employed for peak deconvolution using a Gaussian model in the spectral range of 2800–3800 cm^−1^ (water hydrogen bonding region). The intensities and shifts of each O–H peak were analyzed and plotted for the individual WiS electrolytes and binary mixtures. These trends were then utilized to study the water hydrogen bonding environment and the K-ion solvation structure.

### NMR analysis

NMR spectra were obtained on a Bruker Avance III HD 500 MHz NMR Spectrometer equipped with Broadband Prodigy TCI CryoProbe to inspect the Ac^−^ interactions in WiS systems. 5 mm glass NMR tubes (Fisher Scientific) were charged with 500 μL of the electrolyte samples and a sealed capillary filled with a drop of TMS in pure deuterated DMSO-d6 (99% D) was immersed in it. ^13^C NMR shifts were referenced to DMSO-d6. Data were processed in MestReNova 14.3 64 bit.

### Electrochemical measurements

The electrochemical stability of the WiS electrolyte solutions was assessed through linear sweep voltammetry (LSV) using a BioLogic SP 200 potentiostat at room temperature. A three-electrode cell setup (Pine Research, USA) was utilized, comprising a graphite counter electrode and an Ag/AgCl reference electrode immersed in a 3 M KCl aqueous solution. Glassy carbon and Pt (purchased from Basi), Al (purchased from Sigma-Aldrich), Titanium and Stainless Steel (purchased from StonyLAB) served as the working electrode for LSV. LSV curves were recorded at 1 mV s^−1^ and the onset potentials at which a current of 0.1 mA was observed was selected to define the stability window.

## Data availability

The data supporting this article have been included as part of the ESI.†

## Conflicts of interest

There are no conflicts to declare.

## Supplementary Material

RA-015-D4RA08378D-s001
