# Supplementary material for: Water-in-bisalt electrolytes with mixed hydrophilic and hydrophobic anions for enhanced transport and stability for potassium-ion batteries
Source: RSC Adv. 2025 Jan 2;15(1):94–100. doi: 10.1039/d4ra08378d (PMC11694441; doi:10.1039/d4ra08378d)
Supplement: RA-015-D4RA08378D-s001 [file RA-015-D4RA08378D-s001.pdf]

Supplementary Information (SI) for RSC Advances.
This journal is © The Royal Society of Chemistry 2024

Supporting Information

Water-in-Bisalt Electrolytes with Mixed Hydrophilic and Hydrophobic Anions for Enhanced Transport and Stability for Potassium-ion Batteries

Mukhilan Dhasarathaboopathy, Palani Sabhapathy[a], Burcu Gurkan*

Department of Chemical & Biomolecular Engineering, Case Western Reserve University, Cleveland, Ohio 44106

* Corresponding: beg23@case.edu

Table S1. Physicochemical properties of the KAc/H_2O, KOTf/H_2O and the KAc/KOTf binary mixtures

Solution	Molarity (mol L^{-1})	Salt molar fraction (%)	Density ($g.cm^{-3}$)	Viscosity (mPa.s)	Conductivity ($mS.cm^{-1}$)
1 m KAc	0.9	1.7	1.03	1.45	68.7
10 m KAc	6.4	15.2	1.28	5.78	127.4
20 m KAc	9.1	26.4	1.36	17.13	62.8
30 m KAc	10.8	35	1.42	44.69	27.5
1 m KOTf	0.8	1.7	1.02	1.24	72.4
10 m KOTf	5.1	15.2	1.47	3.82	123.3
18 m KOTf	6.7	24.5	1.64	8.33	78
$K(Ac)_{0.3}(OTf)_{0.7}.3.2H_2O$	7.3	23.5	1.62	10.61	79.7
$K(Ac)_{0.5}(OTf)_{0.5}.2.9H_2O$	8	25.6	1.57	14.04	67.1
$K(Ac)_{0.7}(OTf)_{0.3}.2.78H_2O$	8.5	26.4	1.5	18.28	61.7
**$K(Ac)_{0.9}(OTf)_{0.1}.1.5H_2O$**	10.9	39.4	1.48	87.1	18.07

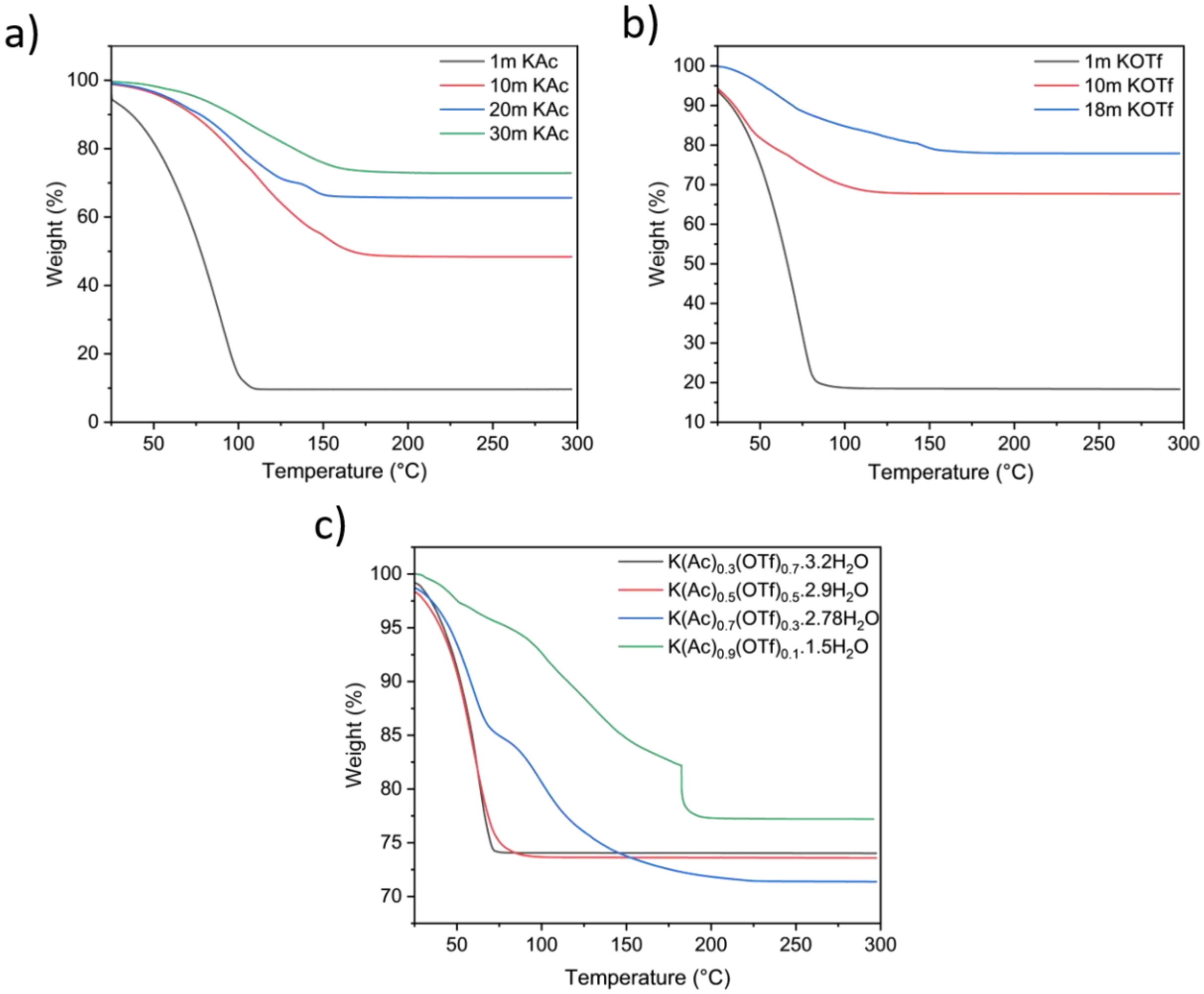

Figure S1. TGA curves of aqueous KAc (a), KOTf (b), and KAc/KOTf binary (c) electrolytes

Table S2. Salt weight percentages of the prepared aqueous samples calculated stoichiometrically and measured experimentally from TGA curves shown in Figure S1.

Sample Solution	Salt weight percentage (%)	
	Calculated (stoichiometric)	Experimental (TGA)
1 m KAc	9.6	9
10 m KAc	48.3	49.5
20 m KAc	65.6	66.2
30 m KAc	72.8	74.6
1 m KOTf	15.8	16.1
10 m KOTf	65.2	67.7
18 m KOTf	77.2	77.9
$K(Ac)_{0.3}(OTf)_{0.7}.3.2H_2O$	73.3	74
$K(Ac)_{0.5}(OTf)_{0.5}.2.9H_2O$	73.2	73.5
$K(Ac)_{0.7}(OTf)_{0.3}.2.78H_2O$	71.4	71.3
$K(Ac)_{0.9}(OTf)_{0.1}.1.5H_2O$	79.4	77.2

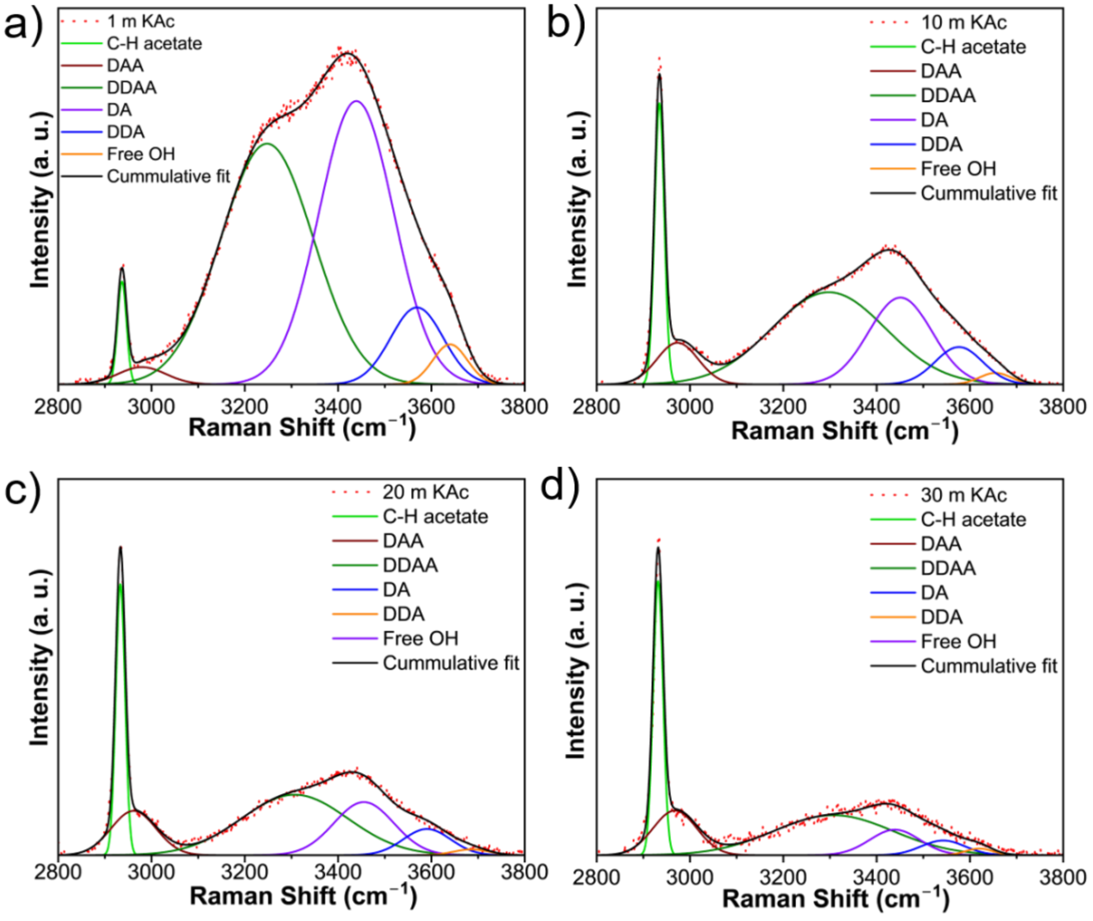

Figure S2. Raman spectra of 1 m KAc (a), 10 m KAc (b), 20 m KAc (c) and 30 m KAc (d) with fitted peaks corresponding to the OH symmetric stretching vibrations of water molecules.

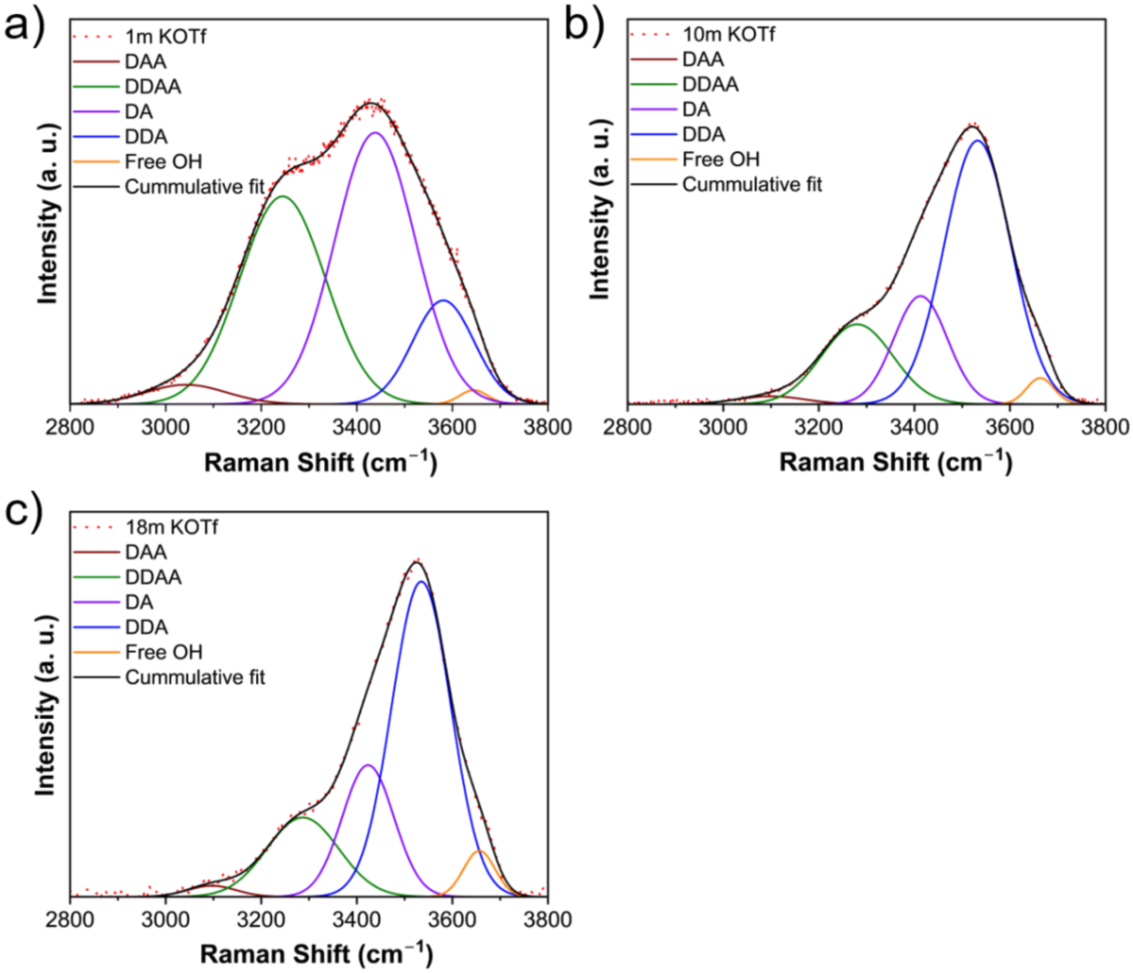

Figure S3. Raman spectra of 1 m KOTf (a), 10 m KOTf (b) and 18 m KOTf (c) with fitted peaks corresponding to the OH symmetric stretching vibrations of water molecules

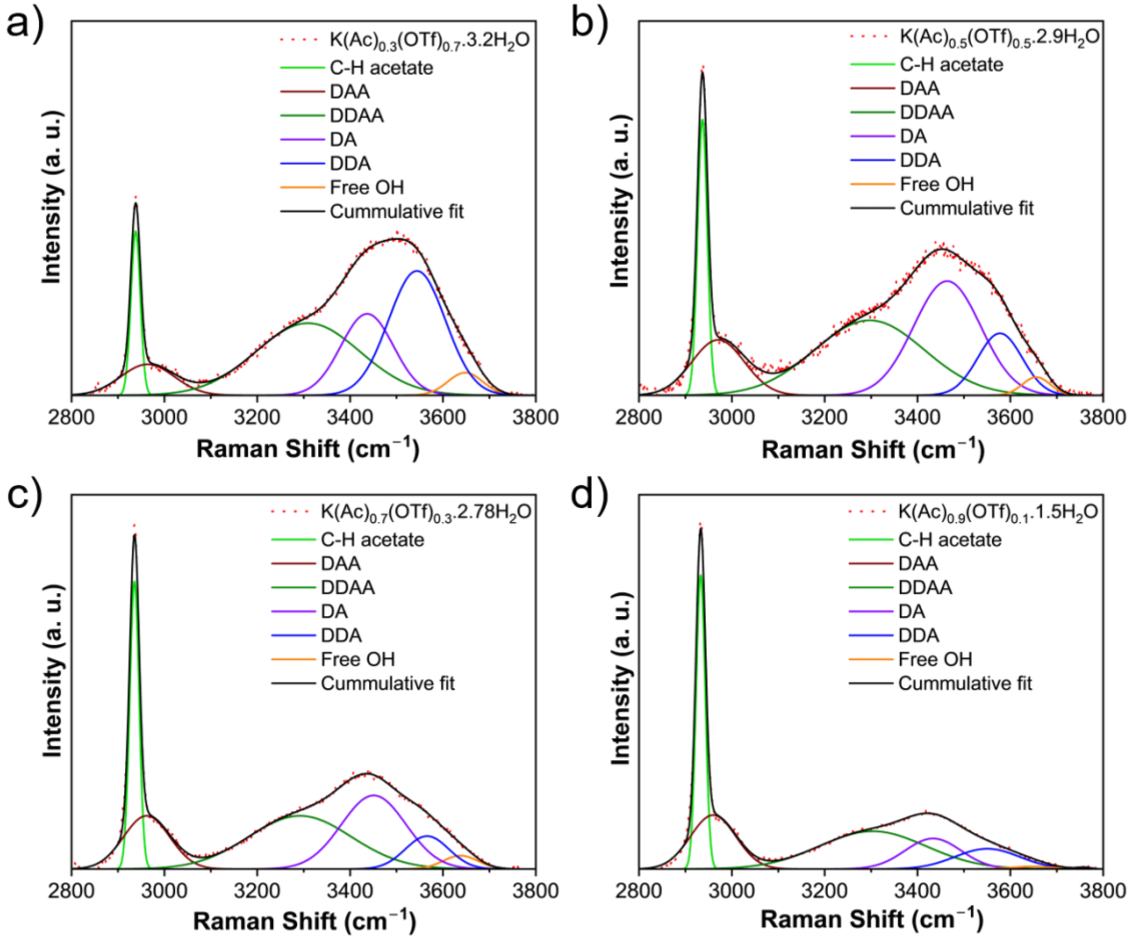

Figure S4. Raman spectra of WiS binary solutions $K(Ac)_{0.3}(OTf)_{0.7}\cdot 3.2H_2O$ (a), $K(Ac)_{0.5}(OTf)_{0.5}\cdot 2.9H_2O$ (b), $K(Ac)_{0.7}(OTf)_{0.3}\cdot 2.78H_2O$ (c), and $K(Ac)_{0.9}(OTf)_{0.1}\cdot 1.5H_2O$ (d) with fitted peaks corresponding to the OH symmetric stretching vibrations of water molecules

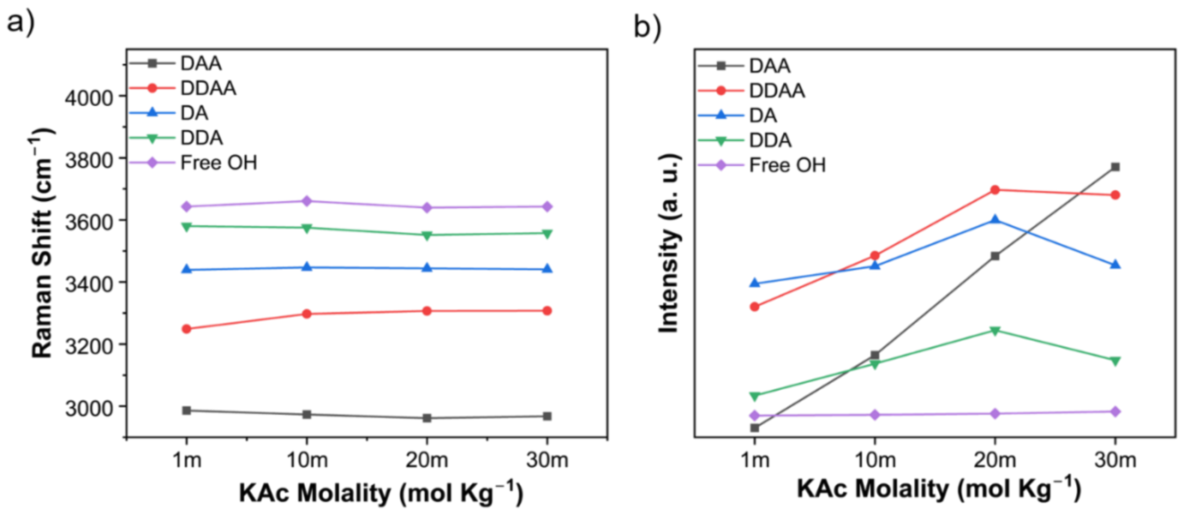

Figure S5. Raman shifts (a) and intensities (b) of DAA, DDAA, DA, DDA, free OH symmetric stretching vibration peaks for KAc electrolytes

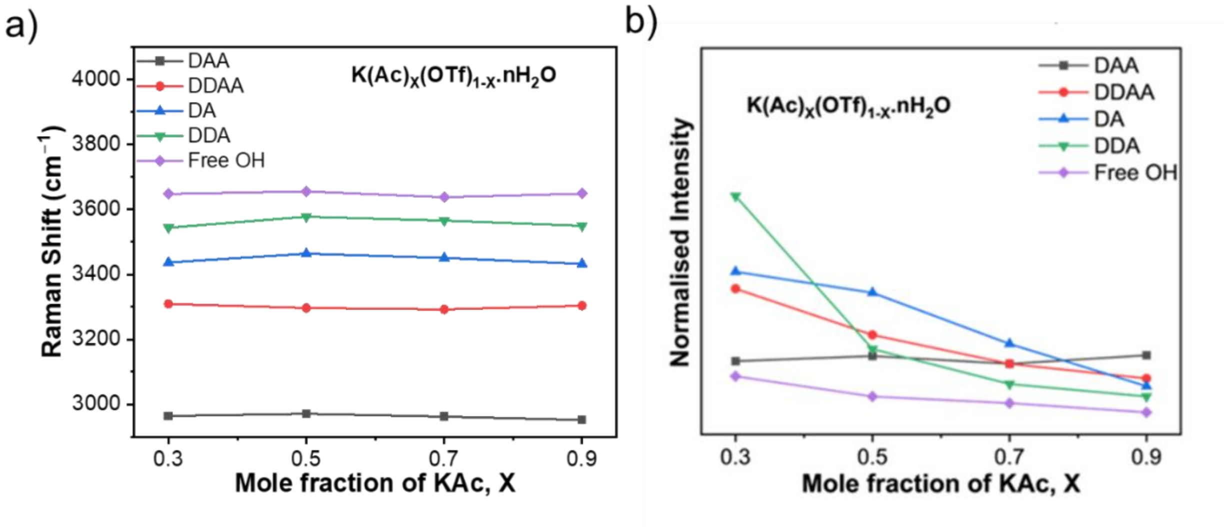

Figure S6. a) Raman shifts of DAA, DDAA, DA, DDA, and free OH symmetric stretching vibrations in WIBS electrolyte as a functional of KAc mole fraction ranging from $K(Ac)_{0.3}(OTf)_{0.7}\cdot3.2H_2O$, $K(Ac)_{0.5}(OTf)_{0.5}\cdot2.9H_2O$ to $K(Ac)_{0.7}(OTf)_{0.3}\cdot2.78H_2O$ to $K(Ac)_{0.9}(OTf)_{0.1}\cdot1.5H_2O$ (32Ac:4OTf); b) Normalized Raman intensity of vibrations due to water

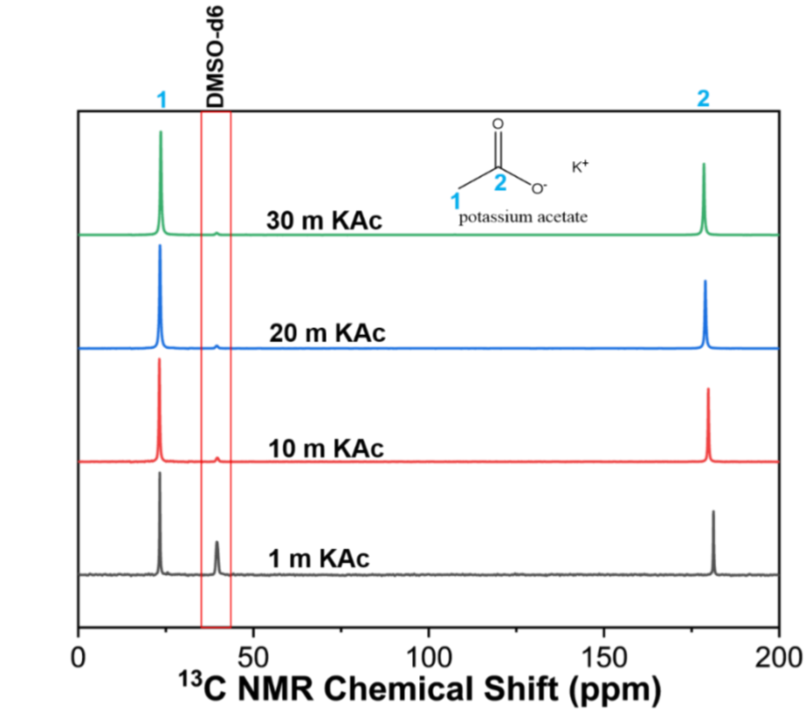

Figure S7. ^{13}C NMR spectra for KAc electrolytes at increasing concentrations

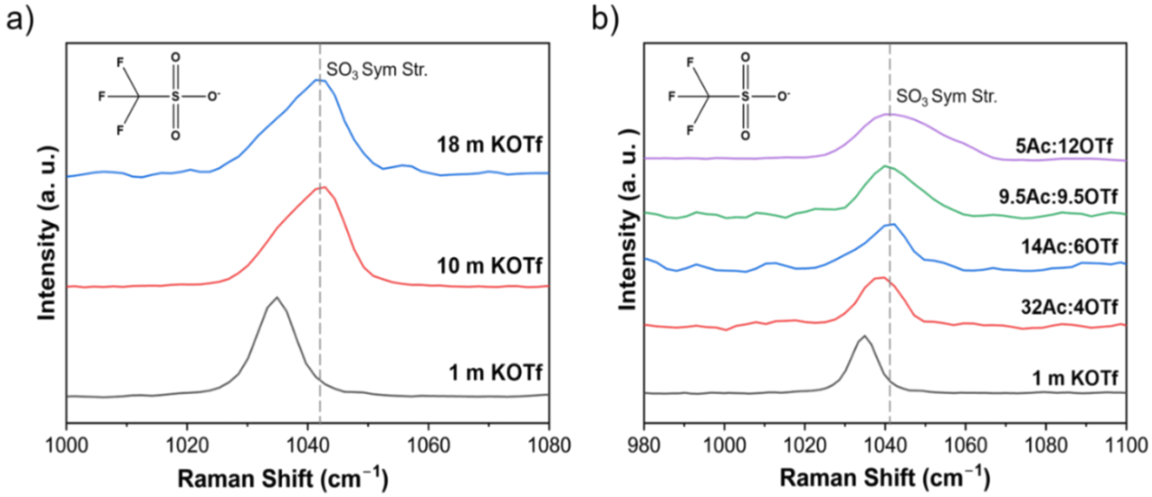

Figure S8. a) Raman peaks of SO_3 symmetric stretching in KOTf with increasing concentrations of OTf; b) Raman peaks of SO_3 symmetric stretching in KOTf for mixtures with increasing concentrations of OTf.

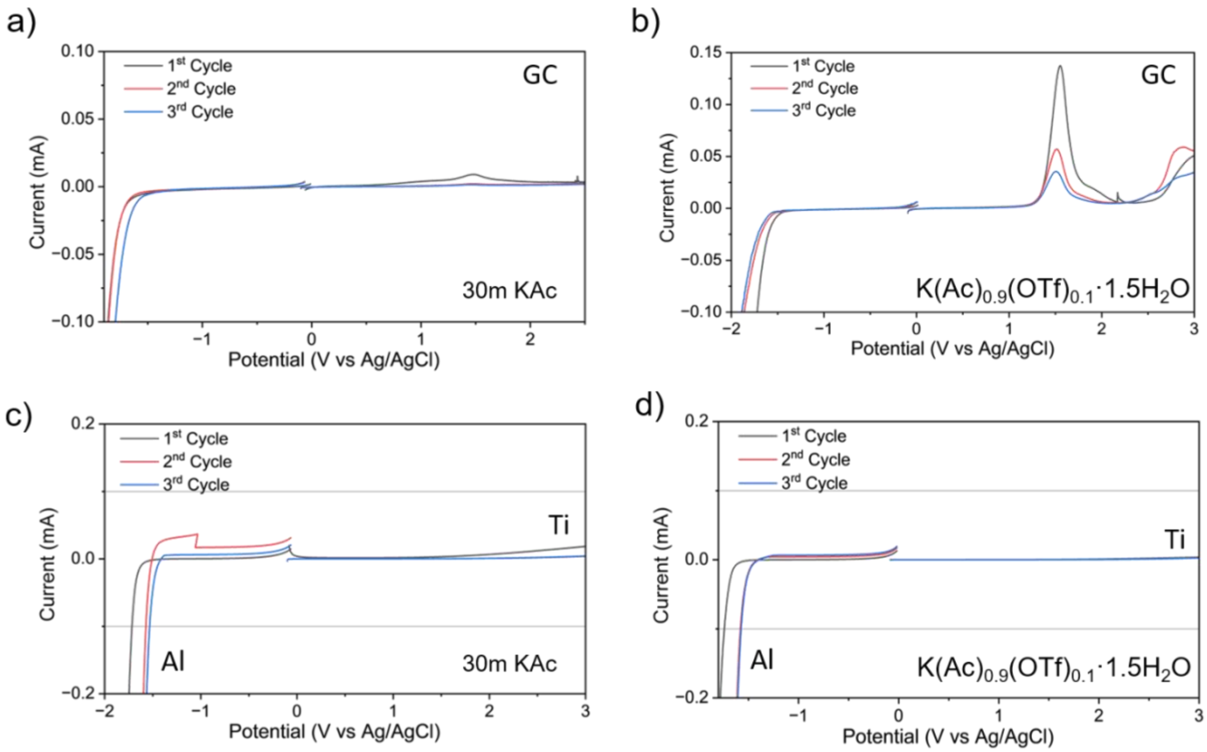

Figure S9. Linear sweep voltammograms of the 1st, 2nd and 3rd cycles for 30m KAc (a and c), and $K(Ac)_{0.9}(OTf)_{0.1}\cdot 1.5H_2O$ (b and d). Panels a and b are with glassy carbon electrode; c and d with aluminum for negative sweep and titanium for positive sweep as marked.
